# The second dimension of the Sforzesco brace correction: analysis of the sagittal shape of the spine

**DOI:** 10.1186/1748-7161-9-S1-O37

**Published:** 2014-12-04

**Authors:** Monia Lusini, Sabrina Donzelli, Salvatore Poma, Nicoletta Trenti, Alice Castelli, Luca Balzarini, Stefano Respizzi, Stefano Negrini

**Affiliations:** 1 ISICO - Italian Scientific Spine Institute, Milan, Italy; 2 University of Milan, Milan, Italy; 3 IRCCS Humanitas, Rozzano, Italy; 4 University of Brescia - IRCCS Don Gnocchi, Milan, Italy

## Background

Scoliosis is a three dimensional deformity, and brace correction should be 3D too. The key role of sagittal and pelvic balance in spinal deformity is very well known, but the effect of brace on sagittal plane remain unknown. The aim of this study is to analyse and compare the sagittal and coronal parameters in a sample of patients treated with Sforzesco Brace.

## Method

Design: observational prospective study on consecutive patients.

Participants: the first 12 female (age 13.07±2.05) patients, with idiopathic scoliosis (41.58°±14.52° Cobb), in Sforzesco brace treatment, who had orthogonal synchronous AP and LL low dose EOS x-rays examination, before treatment start (PRE), with brace (IN) and after four months of treatment at immediate brace take-off (OUT).

## Outcome measures

Sagittal and coronal parameters as automatically calculated by the EOS system.

Statistical analysis: ANOVA for comparisons and Pearson’s coefficient for correlations judged good between 0.5 and 0.8 and high above 0.8.

## Results

Scoliosis worst curves improved statistically IN (12.5°±4.98) and OUT (6.75±3.79) versus PRE.

Pelvic incidence changed significantly comparing IN (45.9±11.6) versus PRE (48.0±10.5) and OUT (48.2±12.3). While Lumbar lordosis (LL) decreased IN, not changing between PRE and OUT, thoracic kyphosis (TK) did not change significantly (LL: PRE=44.4±13.1, IN=35.4±11.6, OUT 43.3±12.5 – TK: PRE=20.9±9.6, IN=18.4±9.8, OUT=19.9±12.8).

Correlations among sagittal parameters increased (normalized) with treatment: out of 21 possible, 13 were good and 2 high at PRE, versus 10 and 7 at IN, and 8 and 10 at OUT respectively.

## Conclusion

Greater attention is needed in the sagittal plane correction made by brace. The Sforzesco brace is able to preserve the sagittal balance of the spine and pelvis, while improving the very well known correlations among sagittal and pelvic parameters. Good braces must preserve and model as much as possible the sagittal balance of the spine and pelvis, without loosing the good correction of the scoliosis deformity. Interestingly, the supposed anatomical (invariant) parameter pelvic incidence showed to change into the brace (IN) versus either PRE and OUT: possible explanations include a sacro-iliac joint movement, even if more data are needed to understand this result.

